# Organic Wastes as Feedstocks for Non-Conventional Yeast-Based Bioprocesses

**DOI:** 10.3390/microorganisms7080229

**Published:** 2019-07-31

**Authors:** Diem T. Hoang Do, Chrispian W. Theron, Patrick Fickers

**Affiliations:** Microbial Processes and Interactions, TERRA Teaching and Research Centre, University of Liège - Gembloux AgroBio Tech, Av. de la Faculté, 2B. B-5030 Gembloux, Belgium

**Keywords:** waste valorization, alternative feedstocks, microbial bioprocesses, value added products, recombinant proteins, yeast biomass, *Yarrowia lipolytica*

## Abstract

Non-conventional yeasts are efficient cell factories for the synthesis of value-added compounds such as recombinant proteins, intracellular metabolites, and/or metabolic by-products. Most bioprocess, however, are still designed to use pure, ideal sugars, especially glucose. In the quest for the development of more sustainable processes amid concerns over the future availability of resources for the ever-growing global population, the utilization of organic wastes or industrial by-products as feedstocks to support cell growth is a crucial approach. Indeed, vast amounts of industrial and commercial waste simultaneously represent an environmental burden and an important reservoir for recyclable or reusable material. These alternative feedstocks can provide microbial cell factories with the required metabolic building blocks and energy to synthesize value-added compounds, further representing a potential means of reduction of process costs as well. This review highlights recent strategies in this regard, encompassing knowledge on catabolic pathways and metabolic engineering solutions developed to endow cells with the required metabolic capabilities, and the connection of these to the synthesis of value-added compounds. This review focuses primarily, but not exclusively, on *Yarrowia lipolytica* as a yeast cell factory, owing to its broad range of naturally metabolizable carbon sources, together with its popularity as a non-conventional yeast.

## 1. Introduction

Pure, ideal sugars, especially glucose, are the main substrates used for biochemical production of chemicals and value-added compounds by microbial cell factories. Because of the important role of glucose in the food industry, however, it is preferential to use alternative carbon sources. In addition to this, concerns over food availability and production, compared with an increasing global population, drive the implementation of recycling and reuse of resources towards waste minimization. Therefore, the utilization of less refined substrates as feedstocks for microbial bioprocesses is an interesting option in this regard. Such feedstocks could potentially also lead to simultaneous reduction of operational costs of these processes, naturally depending on the bioaccessibility of nutrients in the type of feedstock. For example, in lignocellulosic biomass, lignin represents a significant structural barrier to the organisms that are incapable of degrading it, which necessitates harsh pre-treatment (such as high temperature and strong acid or alkali treatment; reviewed by Baruah et al, 2018 [1]) to allow bioaccessibility to other nutrients of the feedstock. In such instances, these pre-treatment steps could thus negate the affordability of the feedstocks. 

**Figure 1:**
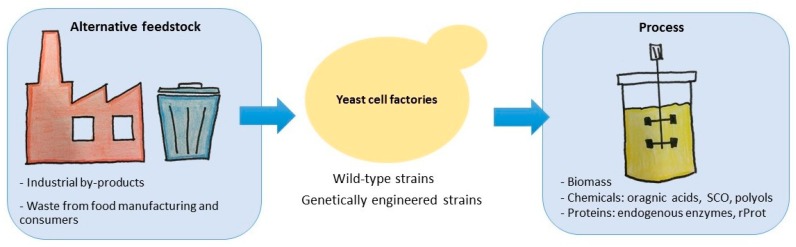
Schematic representation of the principles discussed in this review. Waste materials are used as alternative feedstocks for yeast cell factories, which use them to produce chemicals or proteins of interest.

Non-conventional yeasts (or more accurately ‘non-*Saccharomyces*’ yeasts), such as *Yarrowia lipolytica,* are naturally partially equipped metabolically to hydrolyze and catabolize some of these substrates. For instance, *Y. lipolytica* is able to metabolize hydrophobic substrates (HS) such as alkanes by a specific pathway involving alkane monooxygenases (12 cytochrome P450s encoded by *ALK* genes) [2], fatty-alcohol oxidases as well as dehydrogenases fatty-acyl-CoA synthetases [3]. This yeast is also able to metabolize other HS such as triglycerides and fatty acids owing to the presence of a panoply of genes encoding lipases (mainly *LIP2*, *LIP7,* and *LIP8*; [3] and the six *POX* genes encoding acyl-CoA oxidases involved in β-oxidation [4]). Recent advances in the understanding of these catabolic pathways and in metabolic engineering have allowed genomic engineering to further optimize cell factories to utilize these alternative feedstocks. Recent research articles [5,6] and reviews [7,8] have already focused on such engineering aspects. The goal of this review is to give examples of the utilization of organic wastes as feedstock for the production of valuable chemicals or industrial enzymes by non-conventional yeast species, especially focusing on *Y. lipolytica*, but also relevant examples on others such as *Pichia pastoris* and *Hansenula polymorpha*. The main applications developed are presented according to the main feedstock used (Figure 1).

## 2. Hydrophobic Substrates

As previously stated, *Y. lipolytica* is well known for its ability to degrade HS; therefore, this section is mainly related to this species. Several applications based on the use of HS, mainly triglycerides and fatty acids from various origins, as feedstocks for the synthesis of organic acids derived mainly from the TCA cycle, drug precursors, aroma compounds, and enzymes such as lipases, were described. 

### 2.1. Pure Oil 

Secretion of organic acids from the TCA cycle is one of the characteristic features of *Y. lipolytica*. Organic acids present numerous applications in the industry as food additives, preservatives, antioxidants, or synthons in green chemistry. Among them, citric acid (CA) has been the focus of most research to develop bioprocesses using organic wastes as feedstocks. In fact, the global market for CA is currently around 2.4 million tonnes per year [9,10]. Beside CA, iso-citric acid (ICA) is co-produced at a ratio CA/ICA that depends on the strain considered, the medium composition (mainly C/N balance), and the culture conditions (aeration, pH, iron concentration; [11]). Several research groups have developed strategies and processes to produce CA from hydrophobic substrates while trying to minimize ICA synthesis. Darvishi et al (2009) tested ten different vegetable oils for the production of CA by *Y. lipolytica* strain DSM3286. Although both olive oil and sweet almond oil yielded the highest biomass (8.3 and 8.0 gDCW, respectively), olive oil triggered a higher level of CA productivity (0.006 vs. 0.001 g/g_DCW_.h) and yield (0.36 vs. 0.008 g/g, respectively) [12]. As another example, *Y. lipolytica* UOFSY-1701 grown on sunflower oil (3%) as main carbon source yielded a CA titer of 0.5 g/L after 240h of culture [13]. However, when acetate (10 g/L), a stimulator of the glyoxylate cycle, was added in the culture medium, the CA titer increased substantially to 18.7 g/L [13]. 

On the other hand, ICA is also of interest as it is a useful chiral synthon used in green chemistry [11]. By setting specific culture conditions (pH 6, pO_2_ 0.5–0.6 of saturation, iron-salt concentration 30 mM) for *Y. lipolytica* strain VKMY-2373, ICA was produced predominantly (70 g/L) with an ICA/CA ratio of 1:0.32 using rapeseed oil (concentration of 2%–6%). The ICA productivity and yield were equal to 0.97 g/h and 0.95 g/g, respectively [11]. ICA production was further improved (82.7 g/L, ICA/CA ratio of 1:0.22) by adding oxalic and itaconic acids in the culture medium [14]. In a different approach, based on a genetically engineered derivative of *Y. lipolytica* strain H222 (H222-S4 T1 bearing multiple copies of aconitase genes), ICA was produced with a titer of 56.8 g/L and an ICA/CA ratio of 1:0.42 from 10% of sunflower oil [15]. 

α-ketoglutaric acid (KGA) is another intermediate of the TCA cycle with industrial applications. It is used as the starting material for the synthesis of an antitumor drug, an antioxidative agent, and enhancer of wound healing [16,17]. Under optimal culture conditions (thiamine concentration of 0.063 µg/g_DCW_, pH 3.5 and pO_2_ 0.5 of saturation), *Y. lipolytica* strain VKMY-2412 produced up to 102 g/L of KGA, with productivity and yield of 0.8 g/L.h and 0.95 g/g, respectively, from rapeseed oil (concentration of 2%–6%; [16]). Rapeseed oil was also used to produce succinic acid (SA), which has applications in green chemistry for the synthesis of 1, 4-butanediol, adipic acid, tetrahydrofuran, γ-butyrolactone, and N-methylpyrrolidone. In a medium containing rapeseed oil at a final concentration of 160 g/L, a fed-batch culture of *Y. lipolytica* strain VKMY-2412 in a 5 L bioreactor led to the production of 69 g/L of SA in 156 h [18].

Lipases are enzymes with a broad range of applications as hydrolytic enzymes, as well as for esterification reactions. *Y. lipolytica* is known to produce and secrete large amounts of lipases in the presence of hydrophobic substrates (reviewed in the work of [19]). Mutant strain LgX64.81, obtained by chemical mutagenesis, produced extracellular lipase Lip2p with productivity of 9.9 U/ml.h.A_600_ in a medium containing 0.5% (v/v) of oleic acid [20]. In a 20 L bioreactor, this mutant strain secreted 3044 U/ml and 1300 U/ml of lipase in medium containing olive oil and methyloleate, respectively [21]. Ethyl-oleate and methyl caprylate-caproate were, however, less successful in triggering secreted lipase production, with activities of 195 U/ml and 660 U/ml, respectively [22]. In industrial media containing 3% of methyloleate, a lipase activity of 2010 U/ml was obtained after 96 h of process at 500 L bioreactor scale [22]. Oleic acid was also used in combination with glucose for lipase production in genetically engineered strains of *Y. lipolytica*. A fed-batch culture of strain JMY1105 (a LgX64.81 derivative transformed with a p*LIP2-LIP2* expression cassette) led to a lipase activity of 158.246 U/ml within 80 h [3]. Other oils sourced from almond, hazelnut, and coriander led to low lipase production in *Y. lipolytica* wild-type strain W29, with the highest lipase activity obtained using almond oil after 48 h of culture (2.33 U/ml) [23].

Campesterol, a phytosterol precursor of steroid drugs, was produced from sunflower seed oil using a metabolically engineered derivative of *Y. lipolytica* strain C-22. Through high cell-density fed-batch fermentation, the campesterol titer and productivity was 453 mg/L and 0.008 g/g, respectively, after 120 h of culture [24]. When *Candida (Yarrowia) lipolytica* strain 1094 was grown on corn oil, it accumulated 0.55 (w/w) of lipids per biomass when a pO_2_ of less than 0.05 of saturation was used, while lipid accumulation decreased to 0.37 (w/w) when pO_2_ of less than 0.8 of saturation was used (i.e., lipid accumulation was negatively influenced by increased oxygen availability [25]).

Gasoline and jet fuel contain large quantities of short chain *n*-alkanes such as pentane. Using *Y. lipolytica* strain PO1f derivative disrupted for gene *MFE1* (encoding a multifunctional enzyme) and overexpressing *Gmlox1* (encoding the gene of a lipoxygenase from soybean), linoleic acid could be converted into pentane with titer of 4.98 mg/L [26]. Despite the low conversion yield, this served as a demonstration of the possibility to transform fatty acids into alkanes, compared with the reverse reaction that usually occurs naturally in this yeast. Although not oils, *n*-alkanes have also reportedly been used for the production of CA, ICA, and KGA [11].

### 2.2. Used Oil and Industrial Fats

According to the European biomass industry association (http://www.eubia.org), 40 million tonnes of used cooking oil (UCO) are produced in the European Union (US) each year, while China produces 5 million tonnes/year [27,28]. As the main components of this UCO are triglycerides, it could be used as a feedstock by *Y. lipolytica* to produce compounds such as citric acid, single cell oil (SCO), and lipolytic enzymes. During culture in 10 L bioreactor, *Y. lipolytica* strain SWJ-1b produced 31.7 g/L of citric acid and 6.5 g/L of isocitric acid from 80 g/L of UCO within 336 h of culture [27]. UCO was also successfully used for SCO production using *Y. lipolytica* NCIM 3450, with resultant intracellular lipid content of 0.45 g/g and a lipid production of 2.45 g/L [29]. Using chemical mutagenesis and treatment with cerulenin, a fatty synthase inhibitor, mutants with increased capacity of SCO production were isolated. On medium containing 100 g/L of UCO, one mutant (YlE1) showed a lipid content of 0.55 (g/g) and a lipid productivity of 0.062 g/L.h, representing an almost 50% increase over the parental strain [30].

UCO was also used as a carbon source to coproduce lipase and erythritol. Erythritol is a four carbon polyol produce by osmotolerant yeast that has applications as a sweetener in the pharmaceutical and agro-food industries (reviewed in the work of [31]). With *Y. lipolytica* strain M53 grown in a 5 L bioreactor for 72 h, in a medium containing UCO 3% and ammonium oxalate as nitrogen source at C/N ratio of 87:1, as well as NaCl 80 g/l (used to trigger erythritol synthesis), the maximal lipase activity reached 12.7 U/ml after 24 h, while the final polyol titer was 22.1 g/L, corresponding to a yield of 0.74 g/g [32]. On the basis of a statistical experimental design (Taguchi method), an optimal medium for lipase production containing UCO and arabic gum was formulated. A maximal lipase activity of 12000 U/ml was obtained from a bioreactor culture of *Y. lipolytica* strain W29 using the optimized medium [33]. Moreover, in those conditions, cells accumulated a significant amount of intracellular lipid (0.48 v/v), mainly C_16:0_, C_18:0_, C_18:1_, and C_18:2_ [33]. In a different approach, using a medium containing a mixture of glucose (5 g/L) and UCO 3%, *Y. lipolytica* strain CECT yielded more than 2500 U/ml of lipase activity in seven days [34]. Waste motor oil (WMO) was also used to produce SCO with *Y. lipolytica* NCIM 3450. It yielded to an intracellular lipid content of 0.55 g/g and a lipid production of 0.32 g/L [29].

Animal fat, a by-product of the meat industry, was also used for the production of SCO by *Y. lipolytica* strain ACA-DC50109 [35]. It contained mainly stearin (52%), which, once emulsified with Tween 80 and PEG20000, could be metabolized by yeast cells. For a temperature of 28–33 °C and pH 6, intracellular lipid accumulated to 0.44–0.54 g/g. They were composed of triglycerides (55%) and free fatty acids (35%), of which stearic acid accounts for 80% (w/w). Intracellular unsaturated fatty acids accumulated in response to raw glycerol being added as co-substrate [35]. Pork lard was also used as a feedstock for the production of microbial lipids (up to 0.57 g/g_CDW_) and other value-added metabolites such as lipase (up to 560 U/L) and citric acid (up to 9.2 g/L) [36]. 

γ-decalactone, a fragrant compound with a peachy aroma that is used in the food industry as a flavoring agent, could be obtained by bioconversion of hydrolyzed castor oil or ricinoleic acid using wild-type strain or engineered strain of *Y. lipolytica* either in submerged fermentation (SmF) or solid-state fermentation (SSF). Depending on the system considered, γ-decalactone production ranged from 400 mg/L to more than 10 g/L [37,38,39,40].

### 2.3. Oily Wastewater

Industrial processing of olive oil generates a liquid waste known as olive-mill wastewater (OMW) that contains lipids, sugar, pectins, and polyphenols. Owing to its lipid content, OMW could be used as a feedstock for *Y. lipolytica*. Glucose (65 g/L) was used in combination with OMW to produce citric acid with titer 28.9 g/L, using strain *Y. lipolytica* ACA-DC 50109 [41]. When OMW was blended with crude glycerol (discussed in Section 3), CA titer and yield were 37 g/L and 0.55 g/g, respectively [42]. In those culture conditions, SCO production was 2 g/L with a conversion yield of 0.2 v/v. Extracellular lipase was also produced from OMW by different *Y. lipolytica* isolates (W29, CBS 2073, IMUFRJ50682) with varying success, ranging between 451 and 1041 U/ml of lipase activity [43]; and in a similar study involving 59 *Y. lipolytica* isolates from OMW, diverse lipase activities ranged from 19 U/ml to 2315 U/ml [44].

## 3. Crude Glycerol

Glycerol is a by-product from biodiesel and bioethanol production [45]. It is also a by-product of the saponification process in oleachemical industries. It contains impurities such as methanol, free fatty acids, and inorganic salts; thus rendering its utilization by the chemical industry difficult. According Bagnato et al (2017) the daily crude glycerol production is higher than 80,000 barrels with price lower than 600 USD/ton, providing it with excellent reusability potential for production processes [46]. Glycerol has been demonstrated to be a better carbon source than glucose for *Y. lipolytica* [47] and applications based on the utilization of raw glycerol as feedstock have been investigated [42] and reviewed [48]. Therefore, in this section, we will focus only on recent applications developed for crude glycerol valorization using *Y. lipolytica*. 

KGA and pyruvate (PYR) production by *Y. lipolytica* strain WSH-Z06 obtained by random mutagenesis was investigated in a 3 L fed-batch bioreactor process, in which the glycerol concentration was maintained between 2% and 3%. Under those conditions, the titers of KGA and PYR were equal to 64.7 g/L and 39.1 g/L, respectively, with a final yield of 0.71 g keto-acids /g glycerol [49]. Some genetically engineered strains were also developed for KGA synthesis from glycerol. Overexpression of the genes encoding NADP-dependent isocitrate dehydrogenase (*IDP1*) and pyruvate carboxylase (*PYC1*) led to 19% increased KGA productivity from raw glycerol (12.1 mg/g_CDW_.h) [50].

On the other hand, *Y. lipolytica* strains overexpressing *GUT1* and *GUT2* genes (encoding glycerol kinase and glycerol-3P-dehydrogensae, respectively) were used to produce CA and ICA from crude glycerol [51]. Further, 5 L bioreactor cultures in medium containing 5% (w/v) crude glycerol, the ICA productivity and yield after 96 h were equal to 0.25 g/g and 0.38 g/L.h, respectively, with a total organic acid production of 75.9 g/L [51]. Raw glycerol from a biodiesel production plant served as a feedstock for the synthesis of sodium citrate at 5 L bioreactor scale by *Y. lipolytica* VKM Y-2373, with a titer of 79–82 g/L achieved [52].

More recently, these authors reported the synthesis of PYR with titer of 41 g/L and yield of 0.82 g/g by *Y. lipolytica* VKM Y-2378, also using glycerol from biodiesel production process as a sole carbon source [53]. Cybulski et al (2019) screened 24 *Y. lipolytica* isolates for keto-acid production from glycerol. Among them, strain SKO6 was identified as the best PYR producer with a titer of 99.3 g/L, yield of 0.63 g/g and volumetric productivity of 1.18 g/L.h. In a fed-batch process, with a total glycerol feeding of 200 g/L, PYR titer and productivity were further increased to 125.8 g/L and 0.68 g/g, respectively, using pure glycerol; and to 124.4 g/L and 0.62 g/g, respectively, using raw glycerol from the biodiesel industry. Therefore, remarkably similar production levels could be achieved with the different purities of glycerol feedstock, a promising result for waste valorization [9]. 

An in situ fibrous bed bioreactor (*is*FBB) was used to produce SA from glycerol using a recombinant strain of *Y. lipolytica,* PGC1003, which had the gene *YlSDH5* encoding a subunit of the succinate dehydrogenase complex disrupted [54]. *is*FBB processes allow cells to be immobilized on a solid support, which is known to enhance yeast productivity. Using cotton towel (750 cm^2^, 2 L scale bioreactor) for cell immobilization, SA titer, productivity, and yield equaled 51.9 g/L, 1.46 g/L.h, and 0.42 g/g, respectively [55]. Using the same strain with sugarcane bagasse as the solid support instead, SA production in batch reached 53.6 g/L with an average productivity and yield of 1.45 g/L.h and 0.45 g/g in glucose based medium [56]. The sugarcane bagasse-based *is*FBR was also used to produce 18.9 g/L SA (SA yield of 0.38 g/g) from enzymatic hydrolysate of mixed food waste, in a dual approach to waste valorization [56]. In a fed-batch process involving crude glycerol feeding, SA titer increased up to 209.7 g/L [57].

Glycerol was also used as a feedstock for the synthesis of polyols such as mannitol and erythritol, which were used as sweeteners. Using *Y. lipolytica* strain Wratislavia K1, an erythritol titer of 80 g/L and yield of 0.49 g/g was obtained in the presence of 172 g/L crude glycerol and 2.5% NaCl [58]. For mutants A UV’1 and A-15, up to 27.6 g/l of mannitol was coproduced as a side product. The extracellular and intracellular erythritol and mannitol ratio depended on the purity of the glycerol used (pure or raw) and on the presence of NaCl in the culture medium (used to trigger erythritol production as mentioned earlier) [58]. With repeated batch culture (nutrient replenishment) of strain Wratislavia K1, the production of erythritol increased up to 220 g/L with productivity of 0.54 g/L.h. This repeated batch culture system was tested successfully for 11 cycles, representing more than 1000 h of process time [59]. Strain Wratislavia K1 was also genetically engineered by overexpression of *S. cerevisiae SUC2* and native *GUT1* gene encoding invertase and glycerol kinase, respectively. The resulting strain, which metabolized sucrose and glycerol at a higher rate than the parental strain, was used to produce erythritol from molasses (see Section 4) and crude glycerol. In those conditions, the polyol productivity was 1.1 g/L.h with a yield of 0.67 g/g [60]. A final erythritol titer of 82 g/L was reached after 90 h of culture in a step-wise fed-batch bioreactor culture with molasses (60 g/L) and crude glycerol (150 g/L) as carbon sources [61]. A chemostat culturing process was also developed for *Y. lipolytica* strain MK1 for the conversion of glycerol into erythritol. For a C/N ratio of 80/1, erythritol concentration in the culture medium reached 113.1 g/L with a production rate of 1.1 g/L.h and yield of 0.57 g/g [61]. The chemostat operated at a dilution rate of 0.01 h^-1^ and lasted for 600 h. By developing a push and pull strategy that consisted of overexpressing the genes *GUT1* (encoding glycerol kinase) and *TKL1* (encoding transketolase), an erythritol productivity of 0.073 g/g_DCW_.h was obtained within 96 h using a biomass of 14.6 g_DCW_/L and an initial glycerol concentration of 150 g/L [62]. Glycerol was also used as the main energy source for the conversion of erythritol into erythrulose using a genetically engineered strain overexpressing *EYD1* gene encoding erythritol dehydrogenase [31].

Reports on lipid synthesis and accumulation from pure and raw glycerol are scarce in the literature. In most instances, glycerol is blended with other feedstocks such as HS. *Y. lipolytica* strain UFLA CM-Y9.4 was selected for its ability to grow in the presence of up to 30% of crude glycerol [63]. The strain is able to accumulate up to 63.4% intracellular lipid when grown in the presence of 30 g/L of crude glycerol. The accumulated lipid contained mainly stearic (C_18:0_) and palmitic (C_16:0_) acids. Dobrowolski et al (2016) tested crude glycerol from various industries for the production of lipids by *Y. lipolytica* strain A101. With glycerol from fat saponification, lipid production reached 4.72 g/L in a 5 L bioreactor batch cultivation [64]. Very recently, Sarris et al (2019) screened eleven natural isolates of *Y. lipolytica* for their ability to grow on crude glycerol and to produce metabolites with pharmaceutical and biotechnological interest, notably lipids that could accumulate to levels ranging from 0.33 to 0.84 g/L. Other metabolites were also produced under those conditions, namely citric acid (8.5 to 31.7 g/L), arabinol (2 g/L) and erythritol (0.5 to 3.8 g/L) [42]. γ-decalactone was also produced with titer of 3.5 g/L by *Y. lipolyica* strain CCMA0357 grown in a mixture of raw glycerol and castor oil [65].

The methylotrophic yeast *P. pastoris* also utilizes glycerol efficiently, and this carbon source is commonly used for constitutive recombinant protein (rProt) production. Anastacio et al (2014) investigated in eleven glycerol samples obtained by methanolysis of soybean oil catalyzed by different bases and acids. Crude glycerol containing either KOH or NaOH enhanced cell growth (1.5–2-fold, OD_600_ = 90), with crude glycerol containing NaOH successfully used for heterologous α-amylase production (3.5 U/ml within 30 h) [66]. Alternatively, glycerol can be used as a co-substrate with methanol in rProt production applications based on the methanol-inducible AOX1 promoter, to decrease the amount of methanol added in these processes. In such a strategy, recombinant bovine chymosin was produced by *P. pastoris* using a fed-batch strategy in a 6 L bioreactor with crude glycerol as a co-substrate to methanol, with a maximal biomass yield of 158 g_DCW_/L and a maximal coagulant activity of 192 IMCU/mL after 120 h of induction [67]. Thermostable β-mannanase was produced in *P. pastoris* in a 5 L bioreactor using crude glycerol as main carbon source, resulting in mannanase activity in culture supernatant (2385 U/ml) being only slightly lower (4.2%) than that obtained with pure glycerol [68]. 

Ethanol production by an engineered strain of the methylotrophic, thermotolerant yeast *Ogatea polymorpha*, was achieved from 15% crude glycerol by overexpressing several native and heterologous genes (*PDC1-*pyruvate decarboxylase, *ADH1-*alcohol dehydrogenase*, GCY1-*glycerol dehydrogenase*, DAK1*-dihydroxyacetone kinase, and *FPS1-*glycerol facilitator) [69]. In five days of culture, this strain produced up to 3.55 g/L of ethanol with a productivity and yield of 11.6 mg/g_DCW_.h and 72.3 mg/g, respectively [69].

## 4. Alternative Saccharides

### 4.1. Monosaccharides: Xylose and Galactose

Hemicellulose from plant biomass is one of the most abundant polysaccharides found on earth [6,70,71]. It is mainly composed of xylose, arabinose, mannose, and galactose [70]. The xylose content varies according the plant considered, ranging between 5% and 10% in soft wood (Pine, Spruce), between 12% and 27% in hardwood (Aspen, willow), and up to 35% in agricultural and agro-industrial materials (e.g., Corn cobs) [70]. The three known metabolic routes for xylose catabolism, namely, the Dahms–Weimberg pathway, the xylulose-1P or ribulose-1P pathway, and the xylose isomerase or xylose reductase-xylitol dehydrogenase (XR-XDH) pathway, have been reviewed recently [72]. Several studies reported that *Y. lipolytica* is able to grow on xylose, however, those were in particular conditions with adapted strains or in the presence of helper substrates [26,73]. Cell growth was nevertheless very slow and the final biomass was 16 times lower than that achieved on glucose [73]. Several research groups have attempted to produce microbial lipids from xylose-containing substrates. Using *Y. lipolytica* strain PO1g with detoxified sugarcane bagasse hydrolysate containing xylose, glucose, and arabinose contents of 13.6 g/L, 3.98 g/L, and 2.78 g/L, respectively; resulted in the accumulation of 58% of intracellular lipids. This corresponded to a lipid yield of 6.68 g/L per day and a lipid productivity of 1.76 g per day [74]. In that process, glucose was completely consumed within two days, whereas xylose and arabinose were completely consumed after three and four days, respectively; with a final biomass of 10 g/L produced. Xylose and glucose were found to be catabolized simultaneously, while arabinose was consumed only after glucose depletion in the culture medium [74]. In another study, using the same strain grown on defatted rice bran hydrolysate (containing glucose, xylose and arabinose at contents of 3.16, 1.36 and 0.74 g/L, respectively) led to a maximal lipid content of 0.48 g/g and a lipid yield of 5.16 g/L for a final biomass of 10.75 g/L [75]. 

Engineering strategies were developed to improve the ability of *Y. lipolytica* to grow on xylose. Overexpression of the *XYL1* and *XYL2* genes from *Scheffersomyces (Pichia) stipitis* encoding xylose reductase (Xr) and xylitol dehydrogenase (Xdh), respectively, together with the endogenous xylulokinase (Xk), allowed robust growth on xylose [5]. In fact, the overexpression of native genes encoding XDH and XK derivatives were found to be sufficient to allow *Y. lipolytica* to grow on xylose [6].

A further study used a highly engineered strain overexpressing the native genes encoding XDH, XR, and XK; together with the genes of *DGA2* and *GPD1* from the lipid biosynthetic pathway; along with the deletion of genes *POX1-6* and *TGL4* that are involved in lipid degradation and remobilization. This strain, termed ylXYL + Obese, was used for microbial lipid synthesis from agave bagasse hydrolysate, resulting in a high yield lipid accumulation with lipid content of 67%, at a titer of 16.5 g/l and productivity of 1.8 g/L.h [76]. By combining the heterologous expression of *XYL1* and *XYL2* gene from *S. stipitis* with an adaptive evolutionary engineering step induced by starvation in *Y. lipolytica* strain E26, 15 g/L of intracellular lipid content was accumulated with a productivity of 0.19 g/L.h during 200 h of culture in a 1.5 L bioreactor in medium containing 150 g/L of xylose [77]. 

*Pichia anomala* strain TIB-x229 was used to produce polyols such as D-arabitol (39 g/L), xylitol (30 g/L), and ribitol (8.4 g/L) from xylose (100 g/L), with a global polyol yield of 0.77 g/g [78]. The same strain was used to produce arabitol (28.7 g/L), xylitol (15.7 g/L), and ribitol (15.3 g/L) from waste xylose mother liquor, within 55 h of growth in shake flask. Xylose mother liquor is the left over waste after xylose extraction from sugarcane bagasse or corncob, and is an abundant and cheap feedstock that is difficult to catabolize, as it contains metabolic inhibitors such as furfural and 5-hydroxymethylfurfural (HMF). In the aforementioned study, however, it served as a compatible feedstock. The initial xylose and glucose concentrations in the xylose mother liquor were 90 g/L and 30 g/L, respectively [78].

Using *Pichia stipitis* in an integrated recycle technology (RaBIT) process, ethanol was produced to 30 g/L within 48 h with strain FLP-061 grown on corn stover hydrolysate (starting xylose concentration of 60 g/L, [79]). In the presence of xylose only, ethanol production reached of 20.7 g/L with productivity of 0.17 g/L.h, and when the medium was supplemented with CaCO_3_ and CaCl_2_ (3 g/L) the ethanol production reached 47.4 g/L and 44.8 g/L, respectively; with corresponding productivities of 0.39 g/L.h and 0.37 g/L.h [80]. Using *P. stipitis* ATCC58784 modified by adaptive evolution on corn hydrolysate to increase tolerance toward inhibitors, xylose from acid-treated corncob hydrolysate could be converted to ethanol with productivities ranged from 0.3 to 0.5 g/L.h in the absence and presence of acetic acid, with corresponding yields of 0.4 to 0.43 g/g [81].

*Kluyveromyces marxianus* is able to efficiently produce ethanol from xylose at a high temperature, as this yeast is capable of growth at temperatures higher than most yeasts. Although fermentation using *K. marxianus* strain BUNL-21 led to slightly lower ethanol production at 30 °C compared with *P. stipitis*, the former was capable of growth and ethanol production at 37 °C, and was more tolerant to lignocellulosic by-products commonly toxic to yeasts [82].

Galactose is a constitutive monosaccharide of galactomannans, hemicelluloses, and pectins. A *Y. lipolytica* strain overexpressing the four *GAL* genes of the native Leloir pathway was found able to catabolize efficiently galactose [83]. The resulting strain was used to produce CA and lipids in a 5 L bioreactor in the presence of 6% galactose (C/N 100), resulting in CA titer and yield of 29.2 g/L and 0.51 g/g, respectively. In the same conditions, fatty acids accumulated up to 3.2 g/L with yield of 0.056 g/g. The biomass generated in the process was equal to 19.4 g/L [83].

Defatted rice bran was used as a feedstock for *Y. lipolytica* strain PO1g, after acid hydrolysis (3% sulfuric acid) at 90 °C for 6 h and detoxification to reduce concentrations of the inhibiting compounds 5-hydroxymethylfurfural and furfural [75]. In the resulting hydrolysate, glucose was the predominant sugar (43 g/L) followed by xylose (5 g/L) and arabinose (2 g/L). Starting from a total sugar concentration of 30 g/L, the maximum biomass obtained was 10 g/L with a lipid content of 0.48 and a lipid yield of 5 g/L. Detoxified sugarcane bagasse hydrolysate was also used as a feedstock to grow *Y. lipolytica* strain PO1g. Bagasse hydrolysed with HCl 2.5% generated xylose (14 g/L), glucose (4 g/L), and arabinose (2.3 g/L). Detoxification with Ca(OH)_2_ neutralization lowered the 5-HMF and furfural concentration by 21% and 25%, respectively. After four days of culture of strain Po1g in a medium containing sugarcane bagasse hydrolysate (20 g/L total sugar) and peptone (5 g/L), the maximum lipid content, lipid yield, and lipid productivity grown in were 0.58, 6.7 g/L, and 1.76 g/L-day, respectively [74].

### 4.2. Disaccharides and Polysaccharides

#### 4.2.1. Inulin

Inulin is a polymer consisting of β-(2,1)-linked fructose units attached to a terminal sucrose residue, which accumulate in plant roots and rhizoids [84]. It was first isolated and characterized by the German scientist, Valenti Rose, in 1804 from the roots of flowering plant *Inula helenum* [85]. Inulin can be found in roots of chicory (*Cichorium endivia*), Jerusalem artichoke (*Helianthus tuberosus*), or blue agave (*Agave tequilana*), where it accumulates as a means of energy storage [86]. Inulin, together with oligofructose, is a fructan, a sub-class of carbohydrate characterized by fructosyl–fructose links between monomers. It is used as a sugar substitute in food and stabilizing excipient in pharmaceuticals. Inulin also has a beneficial effect on gut bacteria and is thus used as a prebiotic. Anticancer activity and immuno-modulatory properties have also been reported [85]. The worldwide market of inulin is expected to reach more than 500 million USD by 2025 [87]. This expanding market is generating an increasing amount of inulin-rich waste materials that could be used as a raw carbon source in bioprocesses [86]. The first step of inulin catabolism consists of its hydrolysis into fermentable monosaccharides. This requires the activity of inulinase that are naturally produced by many microogranisms including the yeast species fungi *Kluyveromyces marxianus* and *Pichia guilliermondii,* the fungal species *Penicillium* sp. and *Aspergillus* sp., and bacteria such as *Pseudomonas* sp. and *Clostridium* sp. Endoinulinases and exoinulinases differ by the hydrolysis products, namely inulo-oligosaccharides and fructose, respectively. 

Most non-conventional yeasts are unable to naturally metabolize inulin. Some isolates of *P. guilliermondii* and *Y. lipolytica* have been reported to be endowed with the ability to hydrolyse inulin [88,89], but generally, yeasts require the heterologous expression of the gene encoding exo-inulinase [90]. *Pichia (Ogatea) polymorpha* was reported to possess an inulinase activity induced in the presence of inulin, but repressed in the presence of the reaction products, glucose and fructose [91]. Ethyl-methane sulfonate (EMS)-facilitated mutagenesis of *P. polymorpha* CBS186, however, led to the isolation of mutants able to produce inulinase in the presence of repressing substrates [92].

Various genes encoding inulinase were overexpressed in different yeast genetic backgrounds to introduce the ability to use this substrate as a feedstock, usually with the aim to produce value added chemicals. The *INU1* gene encoding exo-inulinase from *Kluyveromyces marxianus* CBS6556, probably the most popular yeast-derived inulinase, was cloned in a surface display vector and expressed in *Y. lipolytica* SWJ-1b, a CA producer strain [27]. The resulting strain showed an inulinase activity in the range of 15–20kU/gCDW during 120 h of culture in a bioreactor. In those conditions, 79% of the initial amount of inulin was hydrolyzed, yielding 68.9g/L CA and 4.1 g/L ICA, with a final OD_600_ of 30 [93]. A mutated derivative (transformant 30) was constructed by disruption of the gene *ACL* encoding the ATP-citrate lyase enzyme that converts CA into oxaloacetate and acetyl-CoA, combined with the overexpression of *ICL* gene encoding the iso-citrate lyase that catalyzes the conversion of ICA into acetyl-CoA and oxaloacetate [94]. These modifications resulted in significantly increased CA production and significantly reduced lipid content and ICA. From 10% of inulin, a CA titer of 84 g/L was obtained within 214 h, representing an 89.6% yield [95]. When the *INU* gene from *K. marxianus* CBS6432 was overexpressed in *Y. lipolytica* Wratislavia K1 under the control of the strong constitutive *TEF* promoter, (resulting in strain K1 INU6), a CA titer of 105.2 g/L was obtained from 200 g/L of pure inulin in a 235 h fed-batch culture, with an inulinase activity of 14,000 U/g_DCW_ [96]. More recently, the same research group developed a repeated-batch culture process and obtained a CA titer of 200 g/L with strain *Y. lipolytica* mutant strain AWG7 INU8. In their study, they reported a CA productivity of 0.51 g/L.h and a yield of 0.85 g/g [87]. A two-stage process was developed for *Y. lipolytica* K1 INU6 in a 5 L bioreactor to produce erythritol from a mixture of inulin and glycerol. During the first 24 h of culture, the medium contained only inulin (4%) and was then supplemented with glycerol (20%). At the end of the culture (225 h), the erythritol titer was 121 g/L with a yield of 0.6 g/g [96].

As partially described earlier, oleaginous yeasts, such as *Y. lipolytica*, have the ability to produce lipids, oleochemicals, and diesel-like fuels (reviewed in the work of [97]) from different carbon sources. Inulin was also used as a feedstock for fatty acid and SCO production in *Y. lipolytica*. The *INU1* gene was expressed in *Y. lipolytica* strain ACA-DC50109, known for its ability to accumulate significant amounts of lipid [98]. The resulting transformant (Z31) secreted inulinase at a titer of 41.7 U/ml and was able to accumulate 50.6% (w/w) intracellular oil from extract of Jerusalem artichoke tubers in 78 h in a 2 L bioreactor. The accumulated lipids consisted mainly of C_16:0_, C_18:1_, and C_18:2_ [98]. In another study, the exo-inulase gene from *K. marxianus* strain KM-0 and endo-inulinase gene from *Aspergillus niger* strain F4 were co-expressed in *Y. lipolytica* strain ACA-DC 50109 [99]. The enzymes secreted from the resulting transformant (X + N8) acted synergistically to hydrolyze 90% of the inulin, with the inulinase activity reaching 136.6 U/ml in the culture medium. In those conditions, SCO content in the cell was 48%, which corresponds to a production of 6.56 g/L from a starting inulin concentration of 5% (w/v) [99]. The fatty acid in SCO were mainly C_16:0_, C_16:1_, C_18:0_, C_18:1_, and C_18:2_, with C_18:2_ accounting for 53% [99].

As mentioned before, some strains of *P. guilliermondii* such as Pcla22 are able to naturally produce inulinase (11.5 U/ml) [100]. Therefore, this strain was used for the conversion of inulin into SCO in fed-batch fermentation. From a total amount of 7% of inulin, intracellular lipid accumulated up to 60.6% after 96 h of culture with a yield of 0.19 g/g. The fatty acid profile consisted of C_16:0_ and C_18:1_, with the latter accounting for 57.9%. In another study, *P. guilliermondii* M-30 mutant, obtained by UV radiation and LiCl treatments [101], and entrapped in polyvinyl alcohol, was used as a source of inulinase to hydrolyse Jerusalem artichoke extract, with an inulinase activity of 169.3 U/ml after 48h [102]. It is worth mentioning that this cell immobilization allowed significantly increased inulinase activity (28% higher). Inulin hydrolysate was then used in a second step as feedstock for *Rhodotorula mucilaginosa* TJY15a to accumulate SCO (55.6%, w/w) composed of C_16:0_, C_18:1_, and C_18:2_, with C_18:1_ accounting for 50.6% [102].

#### 4.2.2. Molasses (Sucrose)

Molasses is the runoff from sucrose crystallization, and is the main by-product of sugar refineries. It contains mainly sucrose, as well as glucose and fructose, and a lower proportion of nitrogenous compounds. In the EU, 3 million tonnes/year of beet molasse are produced (CEFS Sugar statistic), while China generates 400 million tonnes/year of sugar cane molasse [103]. Isomaltulose, an isomere of sucrose, is used as a sweetener. It can be obtained by direct conversion of sucrose by a sucrose isomerase (SIase). Using a recombinant strain of *Y. lipolityca* S47 expressing the SIase-encoding gene from *Pantoea dispersa* UQ68J under the control of the semi-constitutive hp4d promoter, Wang et al (2019) obtained a isomaltulose production of 102 g/L in shake flask cultures from sugarcane molasses (350 g/L) and corn steep liquor (1 g/L). In fed-batch fermentation at 10 L scale, the isomaltulose titer increased to 161.2 g/L with a yield of 0.96 g/g. At the end of the culture (80 h), all the monosaccharides from molasses were completely consumed, leading to an isomaltulose purity of 97%. At the same time, intracellular lipids accumulated up to 5.3 g/L, which represented 0.43% v/v of the dry cell weight (DCW). They were composed of fatty acids C_14:0_, C_16:0_, C_16:1_, C_18:0_, C_18:1_, and C_18:2_, with C_18:1_ predominating at 45.14% [104].

Wild type strains of *Y. lipolytica* are unable to metabolize sucrose. This is remedied by the heterologous expression of an invertase-encoding gene, usually *SUC2* from S*. cerevisiae*. Therefore, to allow growth on molasses as feedstock, the invertase needs to be expressed constitutively. Using molasses-based broth and the YLY5 recombinant strain expressing genes for both the foreign invertase (*SUC2*) and native lipase (*LIP2*), a lipolytic activity of 2175 U/ml was obtained in a 10 L bioreactor within 96 h [105]. 

Molasses was also used as a feedstock for the production of other recombinant proteins, such as laccases. These enzymes have numerous applications in degradation of xenobiotics, textile dye bleaching, and delignification of lignocellulosic materials. A recombinant *Y. lipolytica* strain Po1g (Suc+) co-expressing the white-rot fungus *Trametes versicolor* laccase IIIb gene was grown in a 5 L bioreactor in molasses-based medium, resulting in laccase productivity of 0.093 U/h and a yield of 0.03 U/g of substrate, with a final activity of 4 U/ml after 50 h [106].

Using *Y. lipolytica* strain JMY4086 metabolically engineered to accumulate intracellular lipids from a sugar beet molasses (245 g/L) and glycerol (different concentrations tested), the highest lipid content obtained was 0.31 g/g_DCW_ [107,108]. When the process was operated in chemostat for 450 h, the lipid productivity was 0.43 g/L.h. Under those conditions, CA started to accumulate after 400 h of culture and reach 80 g/L after 550 h [92]. *Y. lipolytica* Q4 strain (*Δdga1, Δdga2, Δlro1, Δare1*, and *Δmfe1*), expressing *SUC2* and three copies *DGA2* gene encoding acyltransferase, was grown on molasses (8% v/v), leading to the accumulation of more than 30% of lipids for a biomass of 14.4 g/L within 72 h. Further addition of molasses led to an increase of biomass, although without any additional lipid accumulation [109].

Sugar beet molasses blended with crude glycerol was used to produce erythritol with a *Y. lipolytica* A101 derivative that overexpressed *SUC2* from *S. cerevisiae.* Using a two-stage fermentation process based on a molasses batch phase, followed by a stepwise glycerol fed-batch phase, the final titer was 52–114 g/L of polyol. The productivity of that process ranged between 0.58 and 1.04 g/L.h, while the yield ranged between 0.26 and 0.57 g/g [110]. Using a similar culturing strategy, a *Y. lipolytica* Wratislavia K1 derivative that coexpressed *SUC2* from *S. cerevisiae* and innate *GUT1* gene led to a final polyol titer of 100.5 g/L, with productivity and yield of 1.1 g/l.h and 0.67 g/g, respectively [60].

Sugarcane molasses was used in combination with grape pomace extract for the production of bovine chymosin by *Komagataella (Pichia) pastoris* (GS115 derivative) in a 5 L bioreactor, with an activity of 8.5 U/ml reached in 60 h for a biomass of 20 gDCW/L [111]. Glycerol production with titer and yield of 65 g/L and 0.33 g/g, respectively, was reported for *Hansenula (Pichia) anomala* grown on molasses-corn steep liquor-based medium [112]. *Pichia veronae* strain HSC-22 was used for bioethanol production from sugarcane molasses. Under optimized conditions, the maximal bioethanol production in a 10 L batch bioreactor was 32 g/L with a yield of 0.44 g/g [113]. 

#### 4.2.3. Recalcitrant Plant Components

As a major structural component of plants, cellulose is the most abundant organic polymer on earth. As it consists of a chain of hundreds to thousands of linked glucose subunits, it is regarded as an ideal alternative feedstock for microbial bioprocesses, particularly for biofuel production. Complete cellulose catabolism is, however, a complicated process that many organisms cannot achieve. In the context of this review, yeasts are not equipped with all of the necessary enzymes to break cellulose down independently. Certain fungal and bacterial species often contain these enzymes, however, and can thus serve as gene-donors for heterologous expression in yeast species. Complete cellulose degradation requires synergistic activities of three different types of enzymes, namely, endoglucanases (which cleave cellulose polymers at internal positions into smaller fragments), cellobiohydrolases (which cleave a few residues inward from the terminal residues of the fragmented products of endoglucanases), and β-glucosidases (which cleave the further fragmented products of cellobiohydrolases).

Cellobiose is a disaccharide example of the product of cellobiohydrolases and substrate for β-glucosidases, consisting of two β-glucose molecules linked by a β (1–4) bonds. Lane et al., (2015) reported the construction of a *Y. lipolytica* strain able to use cellobiose as sole carbon source [114]. For that purpose, they overexpressed genes encoding the cellodextrin transporter cdt-1 and intracellular β-glucosidase gh1-1 from *Neurospora crassa*. Under nitrogen-limited conditions, the resulting strain was able to produce CA from cellobiose with a yield of 0.37 g/g. Six genes encoding putative β-glucosidase were identified in the *Y. lipolytica* genome, and homologous overexpression of two of them, namely *BGL1* (YALI0F16027g) and *BGL2* (YALI0B14289g), endowed the resultant strain with the ability to degrade cellobiose. The resulting strain was used to produce fatty acid methyl esters (FAME) with a titer of 0.8 g/L [115]. 

Other engineering strategies towards similar means concerned the heterologous expression of endoglucanase and cellobiohydrolase from *Trichodema reesei* (cellulose degradation) [116] or xylanases from *Thermobacillus xylanilyticas* [117], *Trichoderma harzianum* (XynII), and *A. niger* (XlnD) (hemicellulose degradation) [118].

Using a derivative of *Y. lipolytica* strain ΔpoxB12 (MATA, *xpr2-322, pox1-6Δ, pTEF-BGL1, pTEF-BGL2*, [115]), overexpressing genes coding for endoglucanase *EGI* and *EGII* from *T. reesei* and cellobiohydrolases from *Neurospora crassa* (*CBHI*) and from *T. reesei* (*CBHI*), microcrystaline cellulose such as Avicel could be used as a feedstock for *Y. lipolytica* cell growth and product synthesis. Various types of these approaches in *Y. lipolytica* were recently reviewed by Ledesma-Amaro and Nicaud (2016) [5].

In a similar strategy, *P. pastoris* was equipped with the ability to produce and secrete an endoglucanase and β-glucosidase from *A. niger* and a cellobiohydrolase (CBH2) from *T. reesei*. The resultant strain was capable of growth on cellobiose (β-glucosidase substrate) and carboxymethyl cellulose (endoglucanase substrate), but not on Avicell (CBH substrate, but all three enzymes required for growth). The enzyme cocktail in the supernatant was, however, able to hydrolyse Avicell at 50 °C, indicating that the growth temperature for this yeast may have been insufficient for optimal CBH activity [119].

Starch is another plant polymer of glucose, although it is used for energy storage rather than structure, as in the case of cellulose. Starch is formed by amylopectin and amylose chains that cannot be naturally metabolized by *Y. lipolytica*, that is, without the heterologous expression of specific hydrolytic enzymes. Ledesma-Amaro et al., (2015) reported the heterologous expression of α-amylase from *Oryza sativa* and glucoamylase from *A. niger* for the production of intracellular fatty acids from starch. In an optimized medium containing industrial raw starch (N/C ration of 90), intracellular lipid accumulated up to 27% of DCW [120].

## 5. Food Wastes

Approximately 1.3 billion tons of food are wasted annually, an amount equivalent to one-third of the total annual food production [121,122]. Such food wastes are rich in carbohydrates, nitrogen, and fats, and could thus be used as feedstocks for the synthesis of value added compounds using microorganisms. For instance, Li et al (2018) performed an efficient metabolic evolution of *Y. lipolytica* strain PSA02004 and produced SA with a titer and yield of 18.9 g/L and 0.38 g/g, respectively, from mixed food waste hydrolysates [56]. The agrofood industry also generates a huge amount of waste originating from manufacturing processes. The citrus juice industry produces more than 15 million tonnes of peel annually that contains soluble sugars, fiber, protein, and fat; thus constituting an interesting feedstock. From a model solution of hydrolyzed Valencia orange peel, the thermotolerant *Pichia kudriavzevii* KVMP10 produced 54 g/L of ethanol from an initial total sugar concentration of 101 g/L at the optimized temperature of 42 °C [123]. Wilkins et al., 2007a, reported ethanol production of 37 g/L by *K. marxianus* from an initial total sugar content of 90 g/L from orange peels [124]. Food waste leachates from dry anaerobic digesters (carbohydrate content of 25 g/L) were used to produce *Y. lipolytica* biomass with intracellular lipid accumulation of up to 49% on dry weight basis after six days of culture at 30 °C [125]. The authors reported that the lipid accumulation is highly dependent on the leachate composition, particularly the C/N ratio.

Peels of *Ananas cosmosus* (pineapples) were used for simultaneous saccharification and fermentation by crude cellulase and *Pichia stipitis* NCIM3498 entrapped in calcium alginate beads to produce ethanol with titer of 10.9 g/L within 48h of culture [126]. 

Karte et al (2012) performed a comparative study of five *Y. lipolytica* isolates on 15 less common HS feedstocks regarding intracellular accumulation of lipids. These substrates were, among others, chicken feather waste, orange pulp waste, prawn shell waste, or fish waste. SCO accumulation was achieved with varying degrees of success, depending of the waste substrate trialed, with fish waste yielding the highest SCO production of 0.14 (g/g) with *Y. lipolytica* strain NCIM3589 [30]. 

On a different, but remotely related note, Brabender et al (2018) also demonstrated that synthetic urine and real human urine could serve as an interesting alternative to ammonium sulfate as a nitrogen source for lipid production using *Y. lipolytica* strain PO1f [127]. A summary of those feedstocks that have been used for production of metabolites and/or rProt by non-conventional yeast is listed in Table 1.

## 6. Conclusions and Prospects

With the global population constantly increasing, there are concerns about the future availability of environmental resources, causing a more urgent demand for technological innovation towards sustainable processes involving recycling or reuse of materials and waste minimization. One approach is the use of ‘waste’ by-products from various industries as nutrient sources for microbial cell factories. Depending on the composition of the feedstock, this could also potentially reduce operation costs.

This review listed numerous examples of laboratory-scale, proof of concept research on waste valorization processes involving non-*Saccharomyces* yeast species. This group of yeast was selected largely because of broader substrate ranges and higher physiological flexibility of some of the species (compared with *Saccharomyces cerevisiae*). After all, the use of different waste materials as feedstocks requires a range of metabolic capabilities. Among this group of yeasts, *P. pastoris* (mainly for recombinant protein production) and *Y. lipolytica* (for both metabolite and recombinant protein production) have (arguably) received the most attention. *Y. lipoytica* has a particularly impressive range of tolerable substrates, and was thus the main focus of this review.

Although the examples provided had not yet reached pilot or industrial scale, important information was gathered regarding the feasibility of this approach, as well as the variable effects of feedstock composition and purity, and the effect of strain selection and culture conditions on the plasticity of product yields and profiles. These considerations, together with the continually improving tools available for genetic manipulation, open numerous future doors for transforming waste matter into valuable products as a means toward resource sustainability. 

## Figures and Tables

**Table 1:** Summary of feedstocks used for production of metabolites or rProt by non-conventional yeasts. Y.l.: *Y. lipolytica*; P.p.: *Pichia pastoris*; O.p.: *Ogatea polymorpha*; P.g.: *Pichia guilliermondii*; R.m.: *Rhodotorula mucilaginosa;* P.a.*: Pichia anomala;* P.v*.: Pichia veronae;* P.s*.: Pichia stipitis*; P.k.: *Pichia kudriavzevii; K.m.: Kluyveromyces marxianus.* CA: citric acid; ICA: isocitric acid; KGA: α-ketoglutaric acid; PYR: pyruvate, SA: succinic acid; Lip2p: extracellular lipase Lip2p; SCO: single cell oil/intracellular lipids; Man: mannitol; Ery: erythritol, FAME: fatty acid methyl ester; FB: fed-batch bioreactor, *is*FBB: in situ fibrous batch bioreactor; rBatch: repeated batch; Chem: chemostat; SF: shake flask; TS batch: two-stage batch; DCW: dry cell weight.

Source of Feedstocks	Yeast	Products	Process Mode	Yield-/titer (maximal value)	References
**Hydrophobic Substrates**					
Olive and Sweet Almond Oil	*Y.l.* DSM3286	CA	SF	0.006 g/g_DCW_.h-0.36 g/g	[13]
Sunflower (3%)	*Y.l. UOFSY-1701*	CA	SF	0.5 → 18.7 g/L	[13]
Rapeseed oil (2%–6%)	*Y.l.* VKMY-2373	ICA/CA	10L FB	70 g/L (ICA/CA = 1:0.32) 0.97 g/h-0.95 g/g	[11]
Sunflower oil 10%	*Y.l.* H222	ICA/CA	SF	56.8 g/L (ICA/CA = 1:0.42)	[15]
Rapeseed oil (2%–6%)	*Y.l.* VKMY-2412	KGA	10L FB	102 g/L-0.8 g/L.h-0.95 g/g,	[16]
Rapessed oil	*Y.l.* VKMY-2412	SA	10L FB	69 g/L	[18]
Oleic acid 0.5% (v/v)	*Y.l.* LgX64.81	Lip2p	SF	9.9 U/ml.h.A_600_	[20]
Olive oil/ethyl-oleate	*Y.l.* LgX64.82	Lip2p	FB	3044 U/ml	[20,21]
Oleic acid/glucose	*Y.l.* JMY1105	Lip2p	SF	158.246 U/ml	[3]
Seed oils	*Y.l.* WT29	Lip2p	SF	2.33 U/ml	[23]
Seed oils	*Y.l.* C-22	Campesterol	SF, 5L FB	453 mg/L-0.008 g/g	[24]
Linoleic acid	*Y.l.* PO1f	pentane	SF	4.98 mg/L	[26]
Castor oil/ricinoleic acid	*Y.l.* wild-type	γ-decalactone	SF	400 mg/L-10 g/L	[38,39,40,128],
Corn oil	*Y.l.* 1094	SCO	1.5L Batch	0.37 g/g	[35]
**Used Oil and Industrial Fats**					
Used cooking oil / single cell oil	*Y.l.* SWJ-1b	CA/ICA	10L Batch	31.7 g/L CA-6.5 g/L ICA	[27]
UCO	*Y.l.* NCIM 3450	SCO -	SF	0.45 g/g-2.45 g/L	[29]
UCO 3%	*Y.l.* M53	Lip2p	5L Batch	12.7 U/ml-0.74 g/g	[32]
UCO-arabic gum	*Y.l.* W29	Lip2p	Batch	12000 U/ml	[33]
Glucose-UCO	*Y.l.* CECT	Lip2p	SF	2500 U/ml	[34]
Waste motor oil (WMO)	*Y.l.* NCIM 3450	SCO	SF	0.55 g/g-0.32 g/L	[29]
Animal fat	*Y.l.* ACA-DC50109	SCO	1.5L Batch	0.54 g/g	[35]
Pork lard	*Y.l.*	SCO-Lip2p-CA	2L Batch	0.57 g/g_CDW_-560 U/L-9.2 g/L	[36]
OMW	*Y.l.* ACA-DC 50109	CA	SF	37 g/L and 0.55 g/g	[41]
OMW	*Y.l.* (W29, CBS 2073, IMUFRJ50682)	Lip2p	2L Batch	1041 U/ml	[129] [44]
Fish waste	*Y.l.* NCIM3589	SCO	SF	0.14 g/g	[29]
**Crude Glycerol**					
Glycerol (2%–3%)	*Y.l.* WSH-Z06	KGA/PYR	3L FB	64.7 g/L-39.1 g/L	[49]
Crude glycerol 5% (w/v)	*Y.l. GUT1-GUT2*	CA/ICA	5L Batch	0.25 g/g-0.38 g/L.h,	[51]
Raw glycerol	*Y.l.* VKM Y-2373	CA	5L Batch	82 g/L	[52]
Raw glycerol	*Y.l.* VKM Y-2378	PYR	10L FB	41 g/L-0.82 g/g	[53]
Raw glycerol	*Y.l.* SKO6	PYR	FB	124.4 g/L-0.62 g/g	[9]
Raw glycerol	*Y.l.* PGC1003	SA	*is*FBB FB	51.9 g/L-1.46 g/L.h 0.42 g/g-209.7 g/L	[55,57]
Glycerol	*Y.l.* Wratislavia K1	Man/Ery	5L Batch	80 g/L-0.49 g/g	[58]
Sucrose and glycerol	*Y.l.* Wratislavia K1	Ery	rBatch	220 g/L-0.54 g/l.h	[59]
Glycerol	*Y.l.* MK1	Ery	Chem	113.1 g/L-1.1 g/L.h-0.57 g/g	[60]
Raw glycerol and castor oil	*Y.l.* CCMA0357	γ-decalactone	SF	3.5 g/L	[65]
Crude glycerol and methanol-inducer	*P.p.*	Recombinant bovine chymosin	6L FB	192 IMCU/ml	[67]
Pure glycerol	*P.p.*	Mannanase	5 L Batch	2385 U/ml	[68]
Crude glycerol 15%	*O.p.*	Ethanol	SF	3.55 g/L 11.6 mg/g_DCW_.h-72.3 mg/g	[69]
**Inulin**					
Inulin	*Y.l.* SWJ-1b	CA	Batch	68.9g/L CA-4.1 g/L ICA	[93]
Inulin 10%	*Y.l.*	CA	5L Batch	84 g/L-0.89 g/g	[95]
Pure inulin 200 g/L	*Y.l.* Wratislavia K1	CA	5L Batch	105.2 g/L	[96]
Pure inulin	*Y.l.* AWG7 INU8	CA	rBatch	200 g/L-0.85 g/g	[87]
Inulin	*Y.l.* ACA-DC50109	SCO	2L Batch	0.50 g/g	[98]
Inulin 5% (w/v)	*Y.l.* ACA-DC 50109	SCO	SF	0.48 g/g-6.56 g/L	[99]
Inulin 7%	*P.g.* Pcla22	SCO	2L batch	0.19 g/g	[100]
Inulin	*P.g.* M-30 mutant	inulinase	SF	128 U/mL	[101]
Inulin	*R.m.* TJY15a	SCO	2L Batch	0.55 g/g	[98]
Inulin and glycerol	*Y.l.* K1 *INU6*	Ery	5L Batch	121 g/L-0.6 g/g	[96]
**Molasses (Sucroses)**					
Molasses-corn steep liquor	*Y.l.*S47	isomaltulose	SF	102 g/L	[104]
Molasses	*Y.l.*S47	isomaltulose	FB	161.2 g/L-0.96 g/g	[104]
Molasses	*Y. lipo* YLY5 (SUC2, LIP2) and extracellular lipase (LIP2)	Lip2p	10 L Batch	2175 U/ml	[105]
Molasses	*Y.l.* Po1g (Suc+)	laccase	5 L Batch	0.093 U/h-0.03 U/g	[106]
Sugar beet-molasses-glycerol	*Y.l.* JMY4086	SCA/CA	Chem	0.31 g/g_CDW_-0.43 g/l.h 80 g/L	[107,108]
Molasses (8% v/v)	*Y.l.* Q4 strain	SCO	SF	0. 30 g/g	[109]
Sugar beet-molasses-crude glycerol	*Y.l.* A101	Ery	TS Batch	114 g/L-0.57 g/g	[110]
Sugar beet molasses blended with crude glycerol	*Y.l.* Wratislavia K1	polyol	TS Batch	100.5 g/L-0.67 g/g	[60]
Sugarcane molasses-grape pomace extract	*P.p* (GS115 derivative)	bovine chymosin	5 L Batch	8.5 U/ml	[111]
Molasses-corn steep liquor-based	*P.a. anomala*	Glycerol	SF	65 g/L-0.33 g/g	[112]
Sugarcane molasses	*P.v.* HSC-22	Bioethanol	10 L Batch	32 g/L-0.44 g/g	[113]
**Xylose and Galactose**					
Sugarcane bagasse hydrolysate	*Y.l.* PO1g	SCO	SF	58%, 6.68 g/L per day	[74]
Rice branch hydrolysate	*Y.l.* PO1g	SCO	SF	48%-5.16 g/L	[75]
Agave bagasse hydrolysate	*Y.l.*ylXYL + Obese		3L FB	67%-16.5 g/l	[76]
Xylose 150 g/l	*Y.l.*E26	lipid	1.5L Batch	15 g/l-0.19 g/L.h	[77]
Xylose (100 g/L)	*P.a.* TIB-x229	polyols such as D-arabitol, xylitol, ribitol	SF	0.77 g/g	[78]
Waste xylose mother liquor	*P.a.* TIB-x230	Polyols	SF	Arabitol (28.7 g/L), Ribitol (15.3 g/L) and Xylitol (15,7 g/L)	[78]
Corn stover hydrolysat xylose concentration of 60 g/L	*P.s.* FLP-061	ethanol	RaBIT	44.8 g/l-0.39 g/l.h-0.37 g/l.h	[79]
Corn hydrolysate	*P.s.* ATCC58784	ethanol	RaBIT	0.5 g/L.h-0.43 g/g	[79]
Galactose	*Y.l.*	CA/SCO	5L Batch	CA= 29.2 g/L-0.51 g/g; SCO 3.2 g/l-0.056 g/g	[83]
**Recalcitrant Plant Components**					
Cellobiose	*Y.l.*	CA	SF	0.37 g/g	[114]
Cellobiose	*Y.l.*	FAME	1.5L Batch	0.8 g/L	[115]
Starch	*Y.l.*	SCO	SF	27% of DCW	[120]
Defatted rice bran	*Y.l.* PO1g	SCO	SF	48%-5 g/L	[75]
Sugarcane bagasse hydrolysate	*Y.l.* PO1g	SCO	SF	58.5%-1.76 g/L-day	[74]
**Food Wastes**					
Mixed food waste hydrolysates	*Y.l.*PSA02004	SA	isFBB	18.9 g/L-0.38 g/g	[56]
Orange peel	*P.k.* KVMP10	ethanol	SF	54 g/L	[123]
Orange peel	*K.m.*	ethanol	SF	37 g/L	[124]
Food waste leachates	*Y.l.*	SCO	SF	49% of DCW	[125]
Peels of *Ananas cosmosus*	*P.s.* NCIM3498	ethanol	SF	10.9 g/L	[126]
